# Tissue Residues, Hematological and Biochemical Effects of Tilmicosin in Broiler Chicken

**DOI:** 10.1155/2014/502872

**Published:** 2014-04-03

**Authors:** Mossad Elsayed, Ashraf Elkomy, Mohamed Aboubakr, Mohamed Morad

**Affiliations:** Department of Pharmacology, Faculty of Veterinary Medicine, Benha University, Moshtohor, Toukh, Qalyubia 13736, Egypt

## Abstract

The aim of this study was to determine the blood and tissue concentrations profile and effect of tilmicosin on some hematological and biochemical parameters in broiler chicken. Fifty clinically healthy Hubbard chickens were orally administered 25 mg/kg BW of tilmicosin once daily for 5 consecutive days. Tissue residues of tilmicosin in slaughtered healthy chicken could not be detected by microbiological assay in all tested tissues except in lung (at 96 hours) and liver and kidneys (at 72 hours) after last administration. Tilmicosin caused temporary decrease in the RBCs and WBCs counts and has no effect on hemoglobin (Hb) and packed cell volume concentration (PCV). Also, the effect of tilmicosin on some biochemical parameters was as follows: the concentrations of creatinine, uric acid, electrolytes (sodium, potassium, and calcium), glucose, AST, ALT, ALP, and HDL-cholesterol in the serum of treated chicken did not change in response to the repeated oral administration of tilmicosin. There were only a temporary significant decrease in total protein and albumin concentrations and a significant increase in cholesterol and triglycerides concentrations. Chicken must not be slaughtered before 4 days from the stopping of tilmicosin administration. Tilmicosin makes temporary changes on hematological and biochemical parameters in broiler chicken.

## 1. Introduction

Many drugs were extensively used on the national and international levels. Sometimes the uncontrolled use of these drugs reached abuse concentrations, with consequent side effects starting from mild symptoms to teratogenic, mutagenic, and carcinogenic effects and tissue residue.

The macrolide antibiotics are a structurally similar group of primarily bacteriostatic compounds. Most drugs in this class were isolated from soil bacteria of the genus* Streptomyces* [1]. Macrolides are effective against* Mycoplasma* spp. and Gram-positive organisms, such as* Streptococcus* spp. and* Staphylococcus* spp., but are only slightly effective against Gram-negative bacteria [2].

Tilmicosin is a broad-spectrum bacteriostatic macrolide antibiotic synthesized from tylosin for veterinary use only. It has an antibacterial spectrum that is predominantly effective against* Mycoplasma* spp.,* Pasteurella* spp., and various Gram-positive organisms with antimicrobial activity against Gram-positive anaerobic bacteria and Gram-negative respiratory pathogens including* Mannheimia haemolytica* and* Pasteurella multocida* [3]. Tilmicosin is used for the treatment of respiratory tract infections in poultry caused by* Mycoplasma gallisepticum*,* Mycoplasma synoviae*,* Ornithobacterium rhinotracheale*, and* Pasteurella multocida* [4–6].

The aim of this study was to investigate the blood and tissue concentrations profile of tilmicosin following repeated oral administrations in broiler chicken. Also, the effect of tilmicosin on some hematological and biochemical parameters was studied following repeated oral administrations in broiler chicken.

## 2. Materials and Methods

### 2.1. Drug (Tilmicosin)

Tilmicosin was obtained as an oral solution from Kepro Company for veterinary products (Holland) under a trade name (Tilmi 25% oral solution). Each mL contains 250 mg of tilmicosin (as tilmicosin phosphate).

### 2.2. Experimental Chicken

Fifty clinically normal Hubbard chickens of 6 weeks of age weighting about 1500 to 1700 g, each chosen randomly from Qaliobiya poultry farm, were used in this investigation. Chickens were fed on a balanced ration free from antibiotics for two weeks to withdraw any antibiotic residues. Before drug administration, the weight of chickens ranged between 1950 and 2250 g. Each chicken was orally administered 25 mg/kg BW of tilmicosin once daily for 5 consecutive days to determine blood and tissue residues and the effect of repeated oral administration of tilmicosin on hematological and biochemical parameters in chicken. The experiment was performed in accordance with the guidelines set by the Ethical Committee of Faculty of Veterinary Medicine.

Blood samples (5 mL) were taken from each slaughtered chicken (*n* = 5) before the start of experimental period (at day 0) and on days 1, 5, 10, 15, and 20 of the study. Values obtained from blood samples at the beginning of experimental periods (at day 0) were regarded as control data.

After the end of the 5th day of repeated oral administration of 25 mg/kg BW of tilmicosin once daily for 5 consecutive days, 5 chickens were slaughtered at 24, 48, 72, 96, and 120 hours after the last dose. From each slaughtered chicken, kidneys, lung, heart, liver, fat, breast muscles, thigh muscles, and skin were taken for drug assay. All blood samples were collected in sterilized centrifugation tubes and allowed to clot. Serum was separated by centrifugation for 10 minutes at 3000 r.p.m. Sera were kept frozen until assayed.

### 2.3. Analytical Procedures

Tilmicosin in both collected blood and tissue samples was assayed using microbiological method of antibiotic using* Bacillus subtilis* (ATCC 6633) as a test organism for tilmicosin [7]. Standard curves were constructed using antibacterial free serum collected from chicken and distilled water. Six wells, 8 mm in diameter, were cut at equal distances in standard Petri dishes containing 25 mL seeded agar. The wells were filled with 100 *μ*L of either the test samples (serum or tissues) or tilmicosin standards. The plates were kept at room temperature for 2 h before being incubated at 37°C for 18 h. Zones of inhibition were measured using micrometers, and tilmicosin concentrations in the test samples were calculated from the standard curve. The lower detectable limit of the tilmicosin assay was 0.025 *μ*g/mL. Semilogarithmic plots of the inhibition zone diameter versus standard tilmicosin concentrations in serum and distilled water were linear between 0.025 and 50 *μ*g/mL. Two grams of each tissue was minced in test tube with 2 mL distilled water. Mixtures were homogenized in homogenizer, centrifuged at 3000 r.p.m for 10 minutes, and the supernatant fluid of each sample was taken and directly assayed microbiologically for tilmicosin concentration.

### 2.4. Hematological Assay

The counting of red and white blood cells was performed [8]. The concentration of hemoglobin (Hb) was determined [9]. The packed cell volume (PCV) values were determined by using microhematocrit method [9].

### 2.5. Biochemical Assay

The determination of serum biochemical constituents was performed by using ready-made kits from Diamond Diagnostics Company (Egypt), Vitro Scient Diagnostic Company (Egypt), and SPINREACT Company (Spain). The biochemical measurements were performed for estimation of the activities of serum creatinine, uric acid, sodium, potassium, calcium, glucose, total protein, aspartate aminotransferase (AST) and serum alanine aminotransferase (ALT), alkaline phosphatase (ALP), albumin, cholesterol, triglycerides, and high density lipoprotein-cholesterol (HDL-chol).

The data were expressed as (mean ± SEM) and analyzed using SPSS (16) software (SPSS Inc., Chicago, USA) and differences between the averages were examined by Duncan's multiple range test. Mean values within a row with different superscript letters are significantly different (*P* < 0.05).

## 3. Results

**Figure 1:**
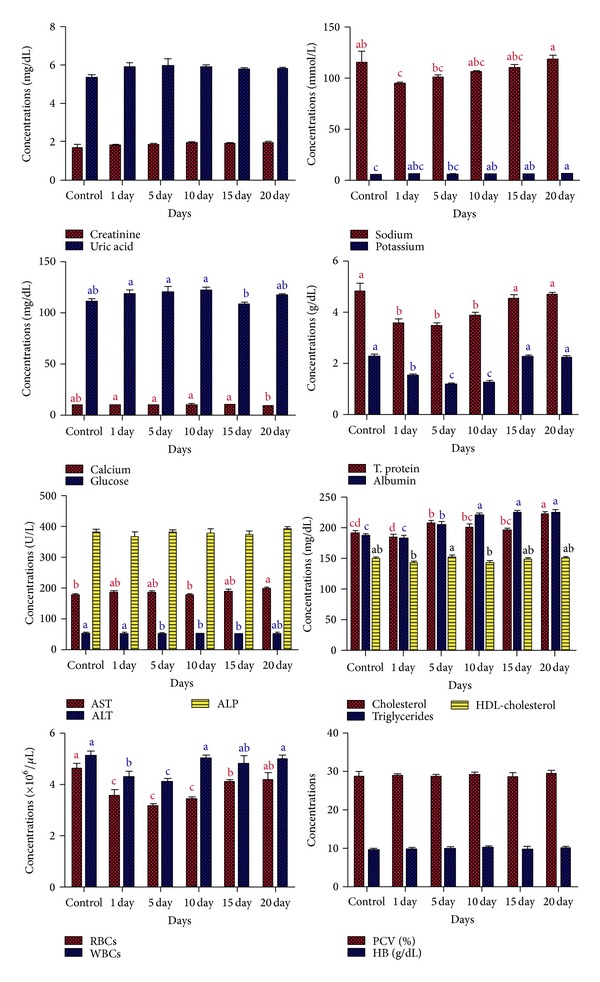
Effect of repeated oral administration of 25 mg/kg BW of tilmicosin daily for five consecutive days on some biochemical and hematological parameters in broiler chicken (*n* = 5).

Blood and tissues concentrations of tilmicosin following oral administration of 25 mg/kg BW once daily for 5 consecutive days in broiler chicken were recorded in Table 1. Tilmicosin could not be detected by microbiological assay in all tested tissues except in lung (at 96 hours) and liver and kidneys (at 72 hours) after last administration. The effect of repeated oral administration of tilmicosin in healthy chicken on RBCs, WBCs count, Hb, and PCV was evaluated in Table 2 and shown in Figure 1. The effects of repeated oral administration of tilmicosin in normal chicken on the biochemical parameters were recorded in Table 3 and shown in Figure 1.

## 4. Discussion

Following repeated oral administration of 25 mg/kg BW of tilmicosin once daily in healthy chicken for 5 consecutive days, the drug could not be detected by microbiological assay in all tested tissues except in lung (96 hours) and liver and kidneys (at 72 hours) after last administration. These results were nearly similar to those obtained after administration of 20 mg/kg BW of tylosin for 5 days to calves and slaughtered at 7 and 14 days after administration and the results showed that, at the 14th day, tylosin levels were lower than the maximal residue limit (MRL) in all target tissues [10]. Also, residue depletion of tilmicosin in broiler chickens was examined after dosing over a 5-day period by incorporation of the drug into drinking water at 37.5 and 75.0 mg/L, and a minimum withdrawal time of 9 days was indicated for residue levels in muscle, liver, and kidney tissues below the maximum residue level [11].

Hematological constituents usually reflect the physiological responsiveness of the animal to its external and internal environments and this is serving as a veritable tool for monitoring animal health. Hematological profile in animals is an important indicator of physiological or pathophysiological status of the body [12]. Tilmicosin caused temporary decreases in the RBCs and WBCs counts and has no effect on hemoglobin (Hb) and packed cell volume concentration (PCV). These obtained results were similar to those recorded after tilmicosin administration and caused statistically significant decrease in RBCs and WBCs counts of rabbits and it achieved at high levels in phagocytes of avian, porcine, and bovine [13, 14]. Also, it was reported that other macrolides might cause similar effect. Azithromycin and clarithromycin decreased RBC and WBC counts in humans [15–17].

Clinical chemical analysis is a fundamental tool used in human and veterinary medicine to diagnose and predict the outcome of disease and to monitor the effect of therapeutic, nutritional, and environmental management [18]. The present study demonstrates that the concentration of creatinine, uric, and electrolytes (sodium, potassium, and calcium) in the serum of treated broiler chicken did not change; these obtained results were similar to those obtained after subcutaneous injection of tulathromycin at a dose of 10 mg/kg BW into rabbits and recorded no change in creatinine, BUN, electrolytes, and glucose concentrations [19]. Also, subcutaneous injection of tilmicosin into mice did not change creatinine, BUN, and glucose concentrations [20]. The concentrations of total protein and albumin in the serum of treated broiler chicken were significantly decreased. These obtained results were similar to the significant decrease in protein and albumin concentrations following subcutaneous injection of tilmicosin into mice [20]. On the other hand, subcutaneous injection of tulathromycin at a dose of 10 mg/kg BW into rabbits did not change both total protein and albumin concentrations [19].

The present study demonstrates that the concentration of liver enzymes (AST, ALT, and ALP) in the serum of treated broiler chicken did not change. These obtained results were similar to those obtained after subcutaneous injection of tilmicosin at a dose of 25 mg/kg BW into New Zealand rabbits and no change in liver enzymes concentrations was observed [13]. Also, intramuscular injection of erythromycin into dogs did not change in AST and ALT enzymes, and only ALP was significantly decreased [21]. Serum ALT activity is a liver-specific enzyme in rabbits [22], and, as serum ALT activity did not change in the present study, it may be suggested that tilmicosin is safe for the liver in previous study [23].

Tilmicosin did not produce changes in high density lipoprotein-cholesterol concentrations in the serum of treated chicken but caused significant increase in cholesterol and triglycerides concentrations. These results were inconsistent with those obtained after subcutaneous injection of tilmicosin into mice and no change in cholesterol concentration was recorded [20]. Also, subcutaneous injection of tulathromycin at a dose of 10 mg/kg BW into rabbits did not change total cholesterol, triglycerides, and high density lipoprotein-cholesterol concentrations [19].

## 5. Conclusions

Chicken must not be slaughtered before 4 days from the stopping of tilmicosin administration. The effect of tilmicosin on hematological parameters was temporary and then returns to normal. Tilmicosin did not make changes in biochemical parameters of treated chicken, only temporary significant decreases in total protein and albumin concentrations and significant increase in cholesterol and triglycerides concentrations.

## Figures and Tables

**Table 1:** Blood (*μ*g/mL) and tissue (*μ*g/g) concentrations of tilmicosin following oral administration of 25 mg/kg BW once daily for 5 consecutive days in broiler chicken (*n* = 5).

Tissues	Time of slaughter after the last dose (hours)				
	24	48	72	96	120
Blood	2.29 ± 0.09	0.91 ± 0.04	—	—	—
Kidneys	3.47 ± 0.09	2.79 ± 0.05	0.86 ± 0.03	—	—
Lung	8.76 ± 0.08	5.38 ± 0.07	3.03 ± 0.04	1.44 ± 0.06	—
Heart	—	—	—	—	—
Liver	4.61 ± 0.07	2.78 ± 0.06	1.05 ± 0.02	—	—
Breast muscles	—	—	—	—	—
Thigh muscles	—	—	—	—	—
Fat	—	—	—	—	—
Skin	—	—	—	—	—

—: not detected.

**Table 2:** Effect of repeated oral administration of 25 mg/kg BW of tilmicosin daily for 5 consecutive days on some hematological parameters in broiler chicken (*n* = 5).

Parameters	Days					
	Control	1	5	10	15	20
RBCs (×10^6^/*μ*L)	4.64 ± 0.19^a^	3.57 ± 0.22^c^	3.15 ± 0.08^c^	3.43 ± 0.07^c^	4.08 ± 0.08^b^	4.19 ± 0.27^ab^
WBCs (×10^6^/*μ*L)	5.12 ± 0.18^a^	4.29 ± 0.23^b^	4.10 ± 0.13^c^	5.02 ± 0.12^a^	4.81 ± 0.32^ab^	4.99 ± 0.17^a^
PCV (%)	28.4 ± 1.54	28.8 ± 0.37	28.6 ± 0.40	29 ± 0.63	28.4 ± 1.17	28.8 ± 0.31
Hb (g/dL)	9.38 ± 0.50	9.65 ± 0.34	9.91 ± 0.49	10.09 ± 0.38	9.73 ± 0.66	10.01 ± 0.31

^a,  b,  c^Mean values having different letters in column differ significantly (*P* < 0.05).

**Table 3:** Effect of repeated oral administration of 25 mg/kg BW of tilmicosin daily for 5 consecutive days on some biochemical parameters in broiler chicken (*n* = 5).

Parameters	Days					
	Control	1	5	10	15	20
Creatinine (mg/dL)	1.67 ± 0.20	1.79 ± 0.03	1.81 ± 0.07	1.90 ± 0.07	1.88 ± 0.05	1.92 ± 0.06
Uric acid (mg/dL)	5.31 ± 0.17	5.89 ± 0.23	5.94 ± 0.39	5.90 ± 0.12	5.78 ± 0.06	5.78 ± 0.08
Sodium (mmol/L)	115.6 ± 10.85^ab^	94.6 ± 0.92^c^	100.6 ± 2.56^bc^	105.8 ± 1.07^abc^	110 ± 3.36^abc^	118.4 ± 4.01^a^
Potassium (mmol/L)	5.15 ± 0.14^c^	5.45 ± 0.13^abc^	5.32 ± 0.20^bc^	5.68 ± 0.06^ab^	5.77 ± 0.06^ab^	5.81 ± 0.13^a^
Calcium (mg/dL)	9.44 ± 0.14^ab^	9.59 ± 0.13^a^	9.75 ± 0.11^a^	9.97 ± 0.24^a^	9.88 ± 0.09^a^	9.01 ± 0.20^b^
Glucose (mg/dL)	111.41 ± 2.61^ab^	118.91 ± 3.96^a^	120.63 ± 5.57^a^	122.39 ± 3.43^a^	107.63 ± 2.86^b^	117.70 ± 1.24^ab^
T. protein (g/dL)	4.80 ± 0.32^a^	3.57 ± 0.15^b^	3.46 ± 0.12^b^	3.87 ± 0.11^b^	4.51 ± 0.16^a^	4.68 ± 0.07^a^
Albumin (g/dL)	2.26 ± 0.09^a^	1.50 ± 0.07^b^	1.18 ± 0.03^c^	1.25 ± 0.06^c^	2.24 ± 0.06^a^	2.22 ± 0.07^a^
AST (U/L)	176.53 ± 3.91^b^	185.54 ± 4.62^ab^	186.1 ± 3.10^ab^	176.49 ± 1.66^b^	188.56 ± 7.06^ab^	197.99 ± 1.29^a^
ALT (U/L)	53.67 ± 0.36^a^	53.45 ± 1.09^a^	51.13 ± 0.36^b^	51.28 ± 0.53^b^	50.60 ± 0.38^b^	52.15 ± 0.69^ab^
ALP (U/L)	381.51 ± 8.70	365.99 ± 15.72	382.30 ± 6.34	378.01 ± 13.46	373.16 ± 11.67	391.50 ± 4.84
Cholesterol (mg/dL)	191.30 ± 4.23^cd^	184.25 ± 5.18^d^	206.58 ± 4.69^b^	201.13 ± 5.15^bc^	195.63 ± 3.62^bc^	221.65 ± 4.27^a^
Triglycerides (mg/dL)	186.48 ± 3.31^c^	181.95 ± 5.76^c^	204.40 ± 5.98^b^	219.98 ± 3.93^a^	223.73 ± 3.91^a^	224.29 ± 5.38^a^
HDL-chol (mg/dL)	150.2 ± 1.62^ab^	141.9 ± 3.18^b^	151.6 ± 3.65^a^	142.42 ± 3.98^b^	147.8 ± 1.83^ab^	150.68 ± 1.11^ab^

^a,  b,  c,  d^Mean values having different letters in column differ significantly (*P* < 0.05).
